# Cardioprotective effect of MMP‐2‐inhibitor‐NO‐donor hybrid against ischaemia/reperfusion injury

**DOI:** 10.1111/jcmm.14191

**Published:** 2019-02-07

**Authors:** Anna Krzywonos‐Zawadzka, Aleksandra Franczak, Agnieszka Olejnik, Marek Radomski, John F. Gilmer, Grzegorz Sawicki, Mieczysław Woźniak, Iwona Bil‐Lula

**Affiliations:** ^1^ Department of Medical Laboratory Diagnostics, Division of Clinical Chemistry Wroclaw Medical University Wroclaw Poland; ^2^ Department of Anatomy, Physiology and Pharmacology, College of Medicine University of Saskatchewan Saskatoon Canada; ^3^ School of Pharmacy and Pharmaceutical Sciences Trinity College Dublin Ireland

**Keywords:** cardioprotection, hybrid, MMP‐2‐inhibitor, nitric oxide donor

## Abstract

Hypoxic injury of cardiovascular system is one of the most frequent complications following ischaemia. Heart injury arises from increased degradation of contractile proteins, such as myosin light chains (MLCs) and troponin I by matrix metalloproteinase 2 (MMP‐2). The aim of the current research was to study the effects of 5‐phenyloxyphenyl‐5‐aminoalkyl nitrate barbiturate (MMP‐2‐inhibitor‐NO‐donor hybrid) on hearts subjected to ischaemia/reperfusion (I/R) injury. Primary human cardiac myocytes and Wistar rat hearts perfused using Langendorff method have been used. Human cardiomyocytes or rat hearts were subjected to I/R in the presence or absence of tested hybrid. Haemodynamic parameters of heart function, markers of I/R injury, gene and protein expression of MMP‐2, MMP‐9, inducible form of NOS (iNOS), asymmetric dimethylarginine (ADMA), as well as MMP‐2 activity were measured. Mechanical heart function, coronary flow (CF) and heart rate (HR) were decreased in hearts subjected to I/R Treatment of hearts with the hybrid (1‐10 µmol/L) resulted in a concentration‐dependent recovery of mechanical function, improved CF and HR. This improvement was associated with decreased tissue injury and reduction of synthesis and activity of MMP‐2. Decreased activity of intracellular MMP‐2 led to reduced degradation of MLC and improved myocyte contractility in a concentration‐dependent manner. An infusion of a MMP‐2‐inhibitor‐NO‐donor hybrid into I/R hearts decreased the expression of iNOS and reduced the levels of ADMA. Thus, 5‐phenyloxyphenyl‐5‐aminoalkyl nitrate barbiturate protects heart from I/R injury.

## 1 INTRODUCTION

Revascularization and restoration of blood flow is a standard therapeutic approach to ischaemia.^1^, ^2^ However, restoration of blood flow to previously ischemic myocardium may also result in ischaemia/reperfusion (I/R) injury.^3^ I/R injury of the heart in turn results in microvascular damage or myocardial stunning.^1^, ^4^, ^5^ Although the complete molecular basis for myocardial injury following I/R is not fully understood, it is known that degradation of contractile proteins by proteolytic enzymes is a major contributor to this process. One of these enzymes is matrix metalloproteinase‐2 (MMP‐2). MMP‐2 degrades intracellular contractile proteins^6^, ^7^ including troponin I (TnI).^8^ MLC type 1 and 2^9^, ^10^ and titin^11^ in hearts subjected to I/R.^9^, ^10^, ^12^, ^13^, ^14^ Furthermore, we found that pharmacological inhibition of MMP‐2 protects the heart from I/R injury.^15^

Recent studies showed that the excessive formation of nitric oxide (NO) and generation of a potent oxidant, peroxynitrite (ONOO^−^), during oxidative stress activates (MMP‐2).^6^, ^13^, ^16^ In contrast, physiological amounts of NO mediate cardioprotection and NO metabolite, nitrite, has been demonstrated to be a potential mediator of remote ischaemic pre‐conditioning.^3^ The levels and bioactivity of NO synthesized from l‐arginine are regulated by constitutive, low‐output isoforms of NO synthase, eNOS (endothelial NOS) and nNOS (neuronal NOS), inducible, high‐output NO synthase (iNOS) and the activity of endogenous inhibitors of NOS such as symmetric (SDMA) or asymmetric dimethylarginine (ADMA).^17^, ^18^, ^19^, ^20^, ^21^, ^22^ There is also evidence that the l‐arginine/NO/ADMA pathway plays an important role in the development of cardiovascular disorders including I/R.^14^, ^17^, ^18^, ^19^, ^20^, ^21^

**Figure 1:**
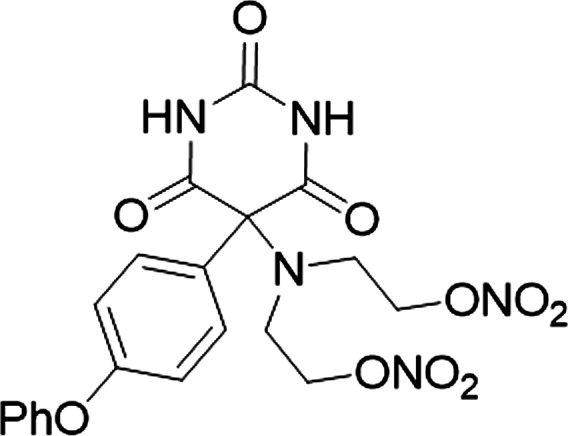
Constitutional formula of 5‐phenyloxyphenyl‐5‐aminoalkyl nitrate barbiturate

In 2012, Wang et al showed that 5‐phenyloxyphenyl‐5‐aminoalkyl nitrate barbiturate hybrid (Figure 1) inhibits the secretion of MMP‐2 and MMP‐9 from cells and this effect depends on the release of NO from this compound.^23^ As changes in the bioactivity of the MMP‐2 and NO pathways may underlie I/R injury, we suggested that the administration of hybrid may normalize the levels of MMP‐2 and NO and ameliorate the impact of I/R on the heart.

We report that this compound has the ability to protect hearts subjected to I/R injury and improve the mechanical function by inhibition of MMP‐2 and restoration of physiological levels of NO.

## 2 MATERIALS AND METHODS

This investigation conforms to the Guide to the Care and Use of Experimental Animals published by the Polish Ministry of Science and Higher Education. This study was approved by the Ethics Committee for Experiments on Animals at the Ludwik Hirszfeld Institute of Immunology and Experimental Therapy Polish Academy of Sciences, Wroclaw, Poland.

### 2.1 Cell culture

The primary Human cardiac myocytes (HCM) from ScienCell Research Laboratories (Carlsbad, CA, USA) were cultured in Dulbecco’s Modified Eagle’s Medium containing Cardiac Myocyte Growth Supplement (ScienCell Research Laboratories), 5% fetal bovine serum, 100 U/mL penicillin, 100 µg/mL streptomycin (all from Sigma‐Aldrich, St. Louis, MO, USA). The cells were cultured at 37°C in a water‐saturated, 5% CO_2_ atmosphere. Cells were passaged at 80% confluence by harvesting with 0.25% trypsin‐EDTA (Sigma‐Aldrich).

### 2.2 The I/R of human cardiac myocytes in culture

Human cardiac myocytes underwent in vitro chemical I/R as proposed in Ref.^24^. Briefly, following 15 minutes aerobic stabilization, the cells were subjected to 9 minutes chemical ischaemia using an inhibitor of cellular respiration and 20 minutes aerobic reperfusion. Cells were subjected to aerobic stabilization and reperfusion in 4‐(2‐hydroxyethyl)‐1‐piperazineethanesulfonic acid (HEPES) buffer (5.5 mmol/L HEPES, 63.7 mmol/L CaCl_2_, 5 mmol/L KCl, 2.1 mmol/L MgCl_2_, 5.5 mmol/L glucose, 10 mmol/L taurine) containing additional 55 µmol/L CaCl_2_ and 0.75 mg/mL BSA. In ischaemia experiments, the cells were subjected to HEPES buffer containing 4.4 mmol/L 2‐deoxyglucose and 4.0 mmol/L sodium cyanide (an inhibitor of electron transport chain). The duration of ischaemia was established in preliminary experiments by measurement of LDH activity released from cells (marker of cell injury). It was determined that 9 minutes of ischaemia was optimal to follow cell recovery during I/R injury (data not shown). At the end of aerobic incubation, the cells were centrifuged for 1 minute at 1500×*g* at RT and the pellet was suspended in the ischaemia buffer and incubated for 9 minutes at RT. Then the buffer was removed by centrifugation at 1500×*g* at RT and the pellet was resuspended in the reperfusion HEPES buffer containing additional 55 µmol/L CaCl_2_ and 0.75 mg/mL mg BSA and incubated for 20 minutes at RT temperature. After reperfusion, the myocytes were centrifuged at 1500×*g* for 5 minutes at RT and the pellet was homogenized and the resultant homogenate stored until assayed at −80°C. In aerobic control experiments, the myocytes were aerobically maintained for the duration of the experiment. In I/R experiments examining the effect of barbiturate, the cells were subjected to I/R in the presence of increasing concentrations of the tested compound (0.1, 1.0 and 10 μmol/L) for 10 minutes before ischaemia and for first the 10 minutes of I/R.

### 2.3 Cell homogenization

Cells were suspended in the homogenization buffer (50 mmol/L Tris‐HCl (pH 7.4) containing 3.1 mmol/L sucrose, 1 mmol/L DTT, 10 µg/mL leupeptin, 10 µg/mL soybean trypsin inhibitor, 2 µg/mL aprotinin and 0.1% Triton X‐100) and homogenized by three cycles of freezing (in liquid nitrogen) and thawing (at 37°C) and then homogenized mechanically (three times for 10 seconds) using a hand‐held homogenizer on ice. Homogenates were centrifuged at 10 000×*g*, for 5 minutes at 4°C and the supernatants were immediately transferred into a fresh tube and stored at −80°C for further biochemical analysis.

### 2.4 MMP‐2, MMP‐9 and iNOS mRNA expression

TRIZOL reagent (Thermo Fisher Scientific, Waltham, MA, USA) was used to isolate total RNA from HCM according to the manufacturer’s instructions. Briefly, 320 ng of pure RNA was reverse transcribed to cDNA using iScript cDNA Synthesis Kit (BioRad, Hercules, CA, USA). Relative RQ‐PCR and CFX96 Touch (BioRad) were used for expression analysis of the following genes: MMP‐2, MMP‐9 and iNOS in a ratio to glyceraldehyde 3‐phosphate dehydrogenase (GAPDH). Briefly, the reaction consisted of iTag Universal Sybr Green Supermix (BioRad), forward and reverse primers (250 nmol/L final conc.), water and cDNA (320 ng) in a final volume of 20 µL. The sequences of primers 5′‐3′ are as follows: iNOSF: CACCTTGGAGTTCACCCAGT′, iNOSR: ACCACTCGTACTTGGGATGC, MMP2F: ATCCAGACTTCCTCAGGCGG, MMP2R: CCTGGCAATCCCTTTGTATGTT, MMP9F: TTGACAGCGACAAGAAGTGG, MMP‐9R: CCCTCAGTGAAGCGGTACAT. The amount of particular mRNAs relative to G6PD was calculated as 2^−ΔCt^. The relative expression of the respective genes was compared in cells that were exposed to aerobic conditions and cells subjected to I/R and I/R with the addition of appropriate concentration of the tested substance.

### 2.5 Langendorff isolated heart perfusion

Wistar rats weighing 300‐350 g were used in these experiments as a surrogate model for analysis of cardioprotection.^24^ The hearts were excised from animals treated with buprenorfin (0.02 mg/kg, ip) and anaesthetized with sodium pentobarbital (40 mg/kg, ip). Spontaneously beating isolated hearts rinsed by immersion in ice‐cold Krebs‐Henseleit Buffer containing 118 mmol/L NaCl, 4.7 mmol/L KCl, 1.2 mmol/L KH_2_PO_4_, 1.2 mmol/L MgSO_4_, 3.0 mmol/L CaCl_2_, 25 mmol/L NaHCO_3_, 11 mmol/L glucose, and 0.5 mmol/L EDTA, pH 7.4, immediately after removal, were suspended on a blunt end needle of the Langendorff system with aorta and maintained at 37°C. Hearts were perfused in the Langendorff system at a constant pressure of 60 mm Hg with Krebs‐Henseleit Buffer at pH 7.4, at 37°C and gassed continuously with 5% carbogen. After stabilization, the hearts were subjected to global, no‐flow ischaemia and then aerobic reperfusion. Coronary flow (CF), heart rate (HR) and left ventricular developed pressure (LVDP) were determined as haemodynamic end‐points of cardioprotection.^25^ Cardiac mechanical function was expressed as the product of HR and LVPD (systolic minus diastolic ventricular pressures)‐rate pressure product at 77 min versus 25 min of perfusion (RPP). Isolated hearts were immediately submerged in liquid nitrogen and stored at −80°C before further analysis.

### 2.6 Global I/R of isolated rat hearts

**Figure 2:**
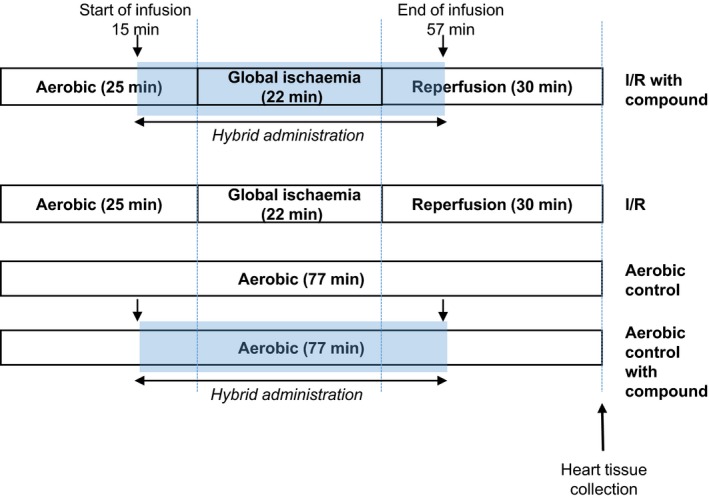
Experimental protocol for I/R and aerobic control with or without hybrid treatment. The hearts were perfused with 5‐phenyloxyphenyl‐5‐aminoalkyl nitrate barbiturate (0.1‐10.0 µmol/L) for the last 10 minutes of aerobic perfusion and the first 10 minutes of reperfusion. Arrows indicate time of start and the end of infusion of the compound. I/R, ischaemia/reperfusion

After 25 minutes of aerobic perfusion, the hearts were subjected to 22 minutes of global, no‐flow ischaemia followed by 30 minutes of aerobic reperfusion. In the study group, following 15 minutes of aerobic perfusion, 5‐phenyloxyphenyl‐5‐aminoalkyl nitrate barbiturate, dissolved in ethanol as a stock solution and then used in final concentrations in the range of 0.1‐10 µmol/L was infused for 10 minutes into the aerobically perfused heart and for 10 minutes at the beginning of reperfusion (Figure 2). Mechanical function of hearts (RPP) was measured at the end of aerobic perfusion (22 minutes) and at the end of reperfusion (75 minutes).

### 2.7 Heart perfusion for cardiomyocytes isolation

The hearts were rapidly excised from rats anaesthetized with sodium pentobarbital (40 mg/kg, ip) as described above. Spontaneously beating hearts rinsed by immersion in ice‐cold Myocyte Isolation Buffer (MIB) containing 120 nmol/L NaCl, 5 mmol/L KCl, 2 mmol/L NaAc, 2 mmol/L MgCl_2_, 1 mmol/L Na_2_HPO_4_, 20 mmol/L NaHCO_3_, 5 mmol/L glucose, 9 mmol/L taurine and 10 mmol/L CaCl_2_ at pH 7.4 immediately after removal were suspended on a blunt end needle of Langendorff system with the aorta and maintained at 37°C. Hearts were perfused in a water‐jacketed chamber of the Langendorff mode at a constant flow of 10 mL/min with MIB buffer containing 1 mmol/L CaCl_2_, pH 7.4, at 37°C, gassed continuously with 5% carbogen for 5 minutes.

### 2.8 Isolation of ventricular cardiomyocytes

After 5 minutes of heart perfusion (in Langendorff system) with MIB containing 1 mmol/L CaCl_2_, the buffer was replaced with MIB containing 5 µmol/L CaCl_2_ and the hearts were perfused for five more minutes as before. The low concentration of CaCl_2_ induced the loss of contractility of cardiomyocytes. After mild swelling of the myocardium with HEPES buffer (120 mmol/L NaCl 140, 5 mmol/L KCl, 2 mmol/L MgCl_2_, 5 mmol/L glucose, 9 mmol/L taurine, 5 mmol/L HEPES) containing 40 µmol/L CaCl_2_, 25 mg of collagenase and 2 mg of protease at pH 7.4, the right ventricle was excised from the heart, rinsed with HEPES buffer containing 100 µmol/L CaCl_2_, 150 mg bovine serum albumin (BSA) and then minced into small pieces in the digestion solution (HEPES buffer containing 100 µmol/L CaCl_2_, 150 mg BSA, 15 mg collagenase and 1 mg protease). The minced tissue was repeatedly digested (six times for 20 and 10 minutes in water bath [37°C]) and 3rd‐6th fraction was used for further experiments.

### 2.9 Chemical ischaemia of isolated ventricular cardiomyocytes

Chemical ischaemia was induced after 15 minutes of treatment with 5‐phenyloxyphenyl‐5‐aminoalkyl nitrate barbiturate (0.1‐10 µmol/L) in HEPES buffer containing 100 µmol/L CaCl_2_, 150 mg BSA by covering the cell pellets with HEPES buffer containing 4 mmol/L 2‐deoxyglucose and 40 mmol/L sodium cyanide The optimal duration of ischaemia, 3 minutes, was established in previous studies.^14^ Three‐minute ischaemia caused approximately 50% loss in cell contractility and viability of the cells was maintained at the level of 70% or higher. After 3 minutes of incubation, the buffer containing sodium cyanide was removed using centrifugation (1 minutes 1500×*g*) and the cell pellet was suspended in the fresh portion of HEPES buffer containing 100 µmol/L CaCl_2_, 150 mg BSA and 5‐phenyloxyphenyl‐5‐aminoalkyl nitrate barbiturate in 0.1‐10 µmol/L concentration. After reperfusion, the cells were centrifuged at 1500×*g* for 5 minutes and the cell pellet, suspended in HEPES buffer (100 µmol/L CaCl_2_, 150 mg BSA), was used for contractility measurement. The aerobic control group was kept exposed to atmospheric air for 38 minutes and the chemical I/R control cardiomyocytes underwent the same experimental protocol without drug treatment.

### 2.10 Measurement of ventricular cardiomyocytes’ contractility

The contractility of cardiomyocytes was measured at the end of the protocol. A 100 µL aliquot of cell suspension was placed in the rapid change stimulation chamber of the IonOptix Contractility System (IonOptix, Milton, MA, USA). After 3 minutes of stabilization, the cardiomyocytes were perfused with oxygenated HEPES buffer containing 2 mmol/L CaCl_2_ (4 mL/min) at 37°C. The cells were continuously paced with 1 Hz and 5 V (MyoPacer; IonOptix) and the contractility expressed as a per cent of peak shortening in comparison to the length of the diastolic cell was measured on an average of five cells per sample. At least five samples per one experimental condition were evaluated.

### 2.11 Preparation of heart homogenates

Hearts previously frozen at −80°C were crushed using a mortar and pestle in liquid nitrogen and then homogenized by sonication in ice‐cold homogenization buffer containing: 50 mmol/L Tris‐HCl (pH 7.4), 3.1 mmol/L sucrose, 1 mmol/L dithiothreitol, 10 mg/mL, leupeptin, 10 mg/mL soybean trypsin inhibitor, 2 mg/mL aprotinin and 0.1% Triton X‐100. The homogenate was centrifuged at 10 000×*g* at 4°C for 15 minutes and the supernatant was collected and stored at −80°C.

### 2.12 Determination of protein concentration

Protein concentration in the cardiac tissue homogenates was determined using Bradford method (BioRad) and BSA (heat shock fraction, ≥98%, Sigma‐Aldrich) served as the protein standard.

### 2.13 MMP‐2 and MMP‐9 protein level in heart homogenates

MMP‐2 and MMP‐9 in hearts homogenates were measured using quantitative the Quantikine ELISA test for Total MMP‐2 and Rat Total MMP‐9 Quantikine ELISA Kit (R&D Systems, Minneapolis, MN, USA), according to manufacturer’s instruction. MMP‐2/MMP‐9 was immobilized with antibody specific to rat MMP‐2 and was detected using anti‐MMP‐2 or anti‐MMP‐9 polyclonal antibody conjugated to horseradish peroxidase (HRP). TMB substrate solution was used to develop the reaction. A minimum detectable dose of the test was as low as 0.033 and 0.013 ng/mL respectively.

### 2.14 Zymography

Gelatin zymography for measurement of MMP activity was performed with the protocol of Heussen and Dowdle and modified by us.^26^ Briefly, 40 µg of heart homogenates were mixed with sample loading buffer (0.5 mol/L Tris‐HCl/0,4% SDS pH 6.8 70% (v/v), glycerol 30% (w/v), SDS 10% (w/v), bromophenol blue 0.012% (w/v), ddH_2_O up to 10 ml) and applied to 8% polyacrylamide gel co‐polymerized with 2 mg/mL of gelatin. After electrophoresis, gels were rinsed in 2.5% Triton X‐100 (3 times for 20 minutes) to remove SDS. Then the gels were washed twice in incubation buffer (50 mmol/L Tris‐HCl pH 7.4, 5 mmol/L CaCl_2_, 150 mmol/L NaCl, 0.05% NaN_3_) for 20 minutes at room temperature. Then the gels were placed in the incubation buffer at 37°C overnight. After digestion, the gels were stained in staining solution (2% Coomassie Brilliant blue G, 25% methanol, 10% acetic acid) for 2 hour and destained (2× for 30 minutes each) in destaining solution (2% methanol, 4% acetic acid). Zymograms were scanned using VersaDoc 5000 (BioRad) and the band intensities were analyzed using Quantity One software v. 4.6.6 (BioRad). MMP activities were expressed as activity per microgram of protein.

### 2.15 Analysis of iNOS by immunoblotting

iNOS tissue expression in heart homogenates was determined using Western Blot. An aliquot of 60 µg of total proteins from heart homogenates was separated on 12% SDS‐PAGE. iNOS transferred on PVDF membrane (Bio‐Rad) was detected with mouse anti‐iNOS polyclonal antibody 1:5000 (Abcam, ab 21775) and secondary goat anti‐mouse IgG horseradish peroxidase conjugate 1:1000 (Bio‐Rad). For protein detection, the ClarityTM Western ECL substrate (Bio‐Rad) was used. ChemiDocTM MP System and Quantity One Software (Bio‐Rad) were used for detection of bands and measurement of their density. iNOS was expressed as AU in ratio to beta‐tubulin (Abcam, ab 108342).

### 2.16 Measurement of endogenic rat asymmetrical dimethylarginine (ADMA)

Endogenous asymmetrical dimethylarginine (ADMA) concentration in rat heart homogenates was assessed using Rat ADMA Elisa Kit was used (Cusabio, Houston, TX, USA). Briefly, the assay is based on competitive inhibition enzyme immunoassay technique. Goat‐anti‐rabbit antibodies were used to bind ADMA from cardiac tissue or labelled ADMA. Then, the antigen was detected by antibodies specific for labelled and unlabelled antigen. ADMA content in tissue homogenates was expressed as ng/mg of total protein.

### 2.17 Nitrate/nitrite assessment

Quantitative Nitric Oxide Assay Kit (Abcam, Cambridge, MA, USA) was used to determine the amount of total nitrate and nitrite in heart homogenates (oxidative products of endogenous NO in the tissue) which served as a measure of NOS activity (NO production), as previously described.^14^ Briefly, the homogenates were diluted 1:1 with deionized water and then deproteinized using centrifugal ultrafiltration. Ultrafiltrates were analysed for total nitrate and nitrite content. In two‐step reaction, the nitrates were converted into nitrites by nitrate reductase and then the nitrites were coupled into a coloured azo compound with maximum absorbance at 540 nm. NO content in hearts was expressed as nmol/L per mg of total protein.

### 2.18 Assessment of LDH activity

Lactate Dehydrogenase Activity Assay Kit (Sigma‐Aldrich) was used to determine the activity of LDH in rat hearts following the manufacturer’s instructions. Briefly, lactate dehydrogenase interconverts pyruvate and lactate with the reduction of NAD to NADH, which is detected with a colorimetric assay at 450 nm. Lactate dehydrogenase served as a marker of cell damage, because of its cytoplasmic location and immediate release into extracellular space due to membrane damage/permeability.

### 2.19 Analysis of MLC1 in heart homogenates

Ventricular isoform of light chain of myosin (MLC1) content in myocardium homogenates was determined using Rat MYL3 ELISA Kit (LSBio, Seattle, WA, USA). Briefly, capture antibodies bind MLC1 from cardiac tissue homogenates. Then, biotin‐conjugated antibodies detected the antigens and avidin‐HRP conjugate with TMB as a substrate allowed for complex visualization. MLC1 in heart tissue was expressed as µg per mg of total protein.

### 2.20 Statistical analysis

GraphPad Prism v. 7 was used to perform the statistical analysis. Statistical analysis was performed with one‐way ANOVA with Tukey’s test as the post‐hoc test or Kruskal‐Wallis and Dunn’s test, as appropriate. Shapiro‐Wilk normality test or Kolmogorov‐Smirnov test was used to assess normality of variances changes. Correlations were assessed using Pearson’s or Spearman’s test, as appropriate. Results were expressed as mean ± SEM *P* < 0.05 was the criterion for statistical significance.

## 3 RESULTS

### 3.1 Effects of 5‐phenyloxyphenyl‐5‐aminoalkyl nitrate barbiturate on mechanical function, coronary blood flow and heart injury

**Figure 3:**
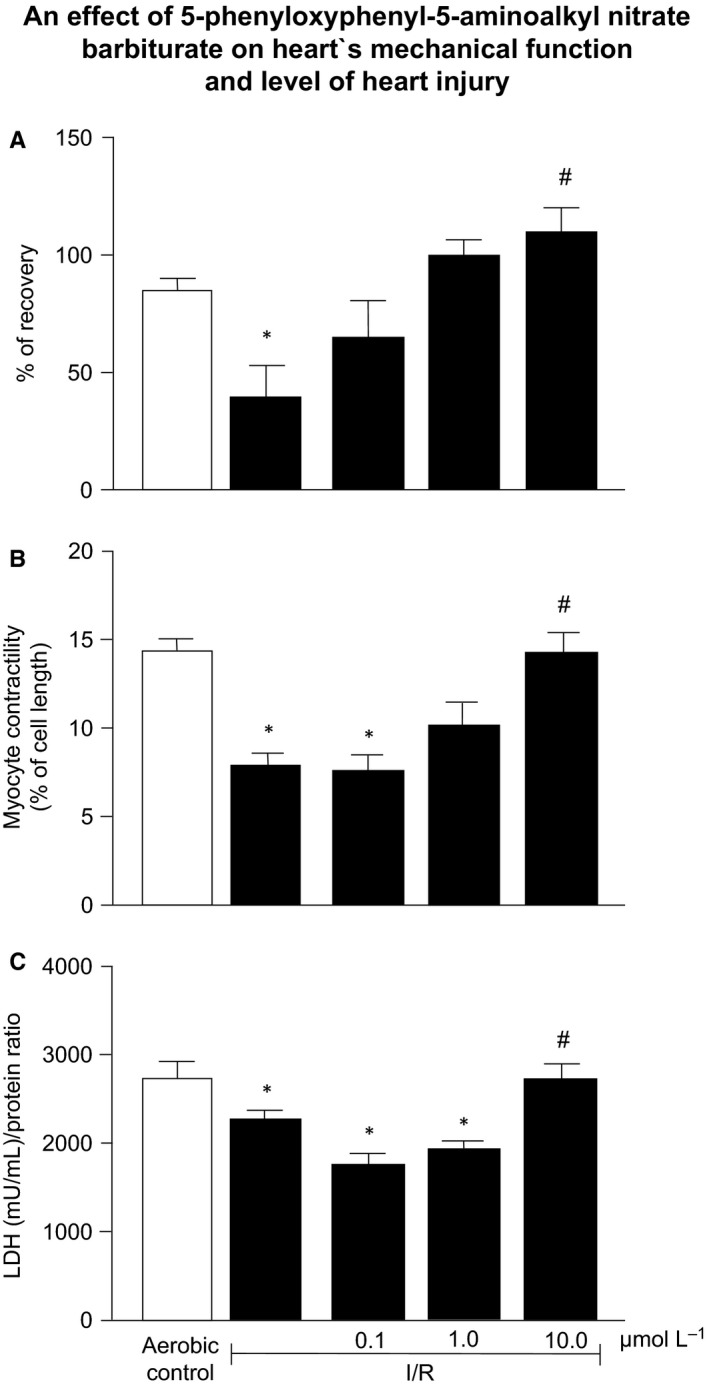
An effect of 5‐phenyloxyphenyl‐5‐aminoalkyl nitrate barbiturate on: (A) recovery of mechanical function of I/R hearts, (B) contractility of I/R cardiomyocytes and (C) LDH level in I/R hearts. *P < 0.05 vs aerobic control; ^#^ *P* < 0.05 vs I/R; mean ± SEM; n = 5‐10

Mechanical heart function, CF, LVDP and HR were significantly decreased in hearts subjected to 22 minutes of global no‐flow ischaemia followed by 30 minutes of reperfusion in comparison to aerobic hearts (Table 1). The 5‐phenyloxyphenyl‐5‐aminoalkyl nitrate barbiturate (1‐10 µmol/L) resulted in concentration‐dependent recovery of mechanical function in comparison to aerobic control (Figure 3A). An infusion of barbiturate also improved the CF and HR.

**Table 1:** An influence of 5‐phenyloxyphenyl‐5‐aminoalkyl nitrate barbiturate on heart rate, LVDP and coronary flow of isolated rat hearts

Parameter	Groups					*P*‐value^d^
	Aerorobic	I/R	Dinitrates (µmol/L)			
			0.1	1.0	10	
HR (bpm)^a^	225.6 ± 24.4	85.2 ± 54.3*	229.3 ± 8.1^#^	278.0 ± 20.2^#^	255.3 ± 27.1^#^	0.0020
LVDP (mm Hg)^a^	83.6 ± 6.4	34.3 ± 12.8*	43.0 ± 19.3	48.6 ± 15.6	47.9 ± 9.6	0.0497
CF (mL/min)^b^	11.6 ± 1.2	2.7 ± 4.2*	11.4 ± 2.6^#^	9.2 ± 2.0^#^	11.9 ± 1.3^#^	0.0005
Recovery (%)^c^	85.2 ± 4.5	39.4 ± 12.7*	65.1 ± 14.8	98.4 ± 7.8^#^	109.5 ± 10.1^#^	0.0014

Mean ± SEM.

a After I/R (77 minutes of the experiment).

b After ischaemia (first minute of reperfusion).

c The difference between RPP in 25 and 77 minutes of the experiment.

d ANOVA test; *P < 0.05 vs Aerobic; ^#^ *P* < 0.05 vs I/R20; n = 5‐10; Aero‐aerobic control; I/R20‐ischaemia/reperfusion control.

Rat cardiomyocytes contractility, expressed as a peak shortening (% of cell length), was significantly decreased (>57%) in cells subjected to in vitro I/R in comparison to cardiomyocytes subjected to aerobic conditions (Figure 3B). Barbiturate treatment protected cell contractility in a concentration‐dependent manner. 5‐phenyloxyphenyl‐5‐aminoalkyl nitrate barbiturate at 10 µmol/L concentration resulted in the full protection of contractility.

Figure 3C shows that changes in LDH content (a marker of cellular injury) in the myocardial tissue during I/R The barbiturate treatment led to concentration‐dependent decrease in LDH release from the heart.

### 3.2 An influence of MMP‐2‐inhibitor‐NO‐donor hybrid on expression of MMP‐2, MMP‐9 and iNOS genes

**Figure 4:**
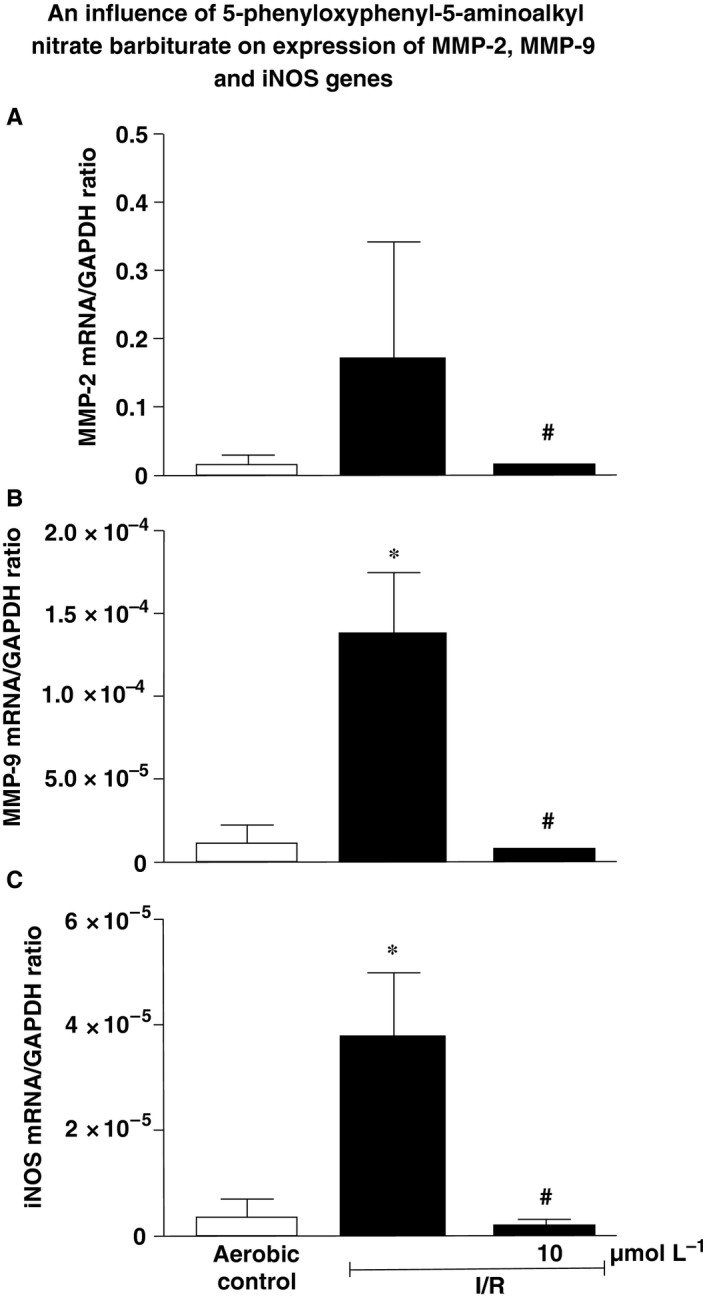
An influence of 5‐phenyloxyphenyl‐5‐aminoalkyl nitrate barbiturate on expression of MMP‐2 (A), MMP‐9 (B) and iNOS (C) genes is human cardiomyocytes in a ratio to GAPDH. iNOS, inducible nitric oxide synthase; GAPDH, glyceraldehyde 3‐phosphate dehydrogenase; I/R, ischaemia/reperfusion; MMP, matrix metalloproteinase; *P < 0.05 vs aerobic control; ^#^ *P* < 0.05 vs I/R; mean ± SEM; n = 5‐10

To assess if the hybrid affects expression of MMP‐2 and MMP‐9 as well as iNOS genes, specific mRNA and corresponding protein expression in cardiomyocytes subjected to I/R have been assessed. Data showed significantly elevated (12‐15 times) expression of MMP‐2, MMP‐9 as well as iNOS genes in comparison to cells maintained in aerobic conditions. Administration of the MMP‐inhibitory barbiturate‐nitrate hybrid led to reduced expression of above enzymes (Figure 4A‐C).

### 3.3 MMP‐2‐inhibitor‐NO‐donor hybrid affects synthesis and activity of MMP‐2 and MMP‐9 in cardiac tissue

**Figure 5:**
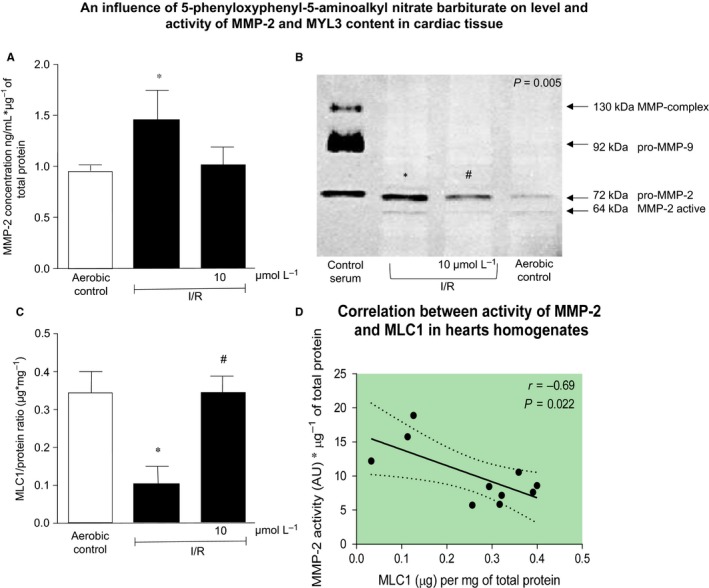
Reduction of MMP‐2 synthesis and activity during infusion of 5‐phenyloxyphenyl‐5‐aminoalkyl nitrate barbiturate into rat heart (A‐B). Protective role of tested drug on MLC1 content in rat hearts (C). Correlation of MMP‐2 activity and MYL tissue content in rat hearts (D). MMP‐2 activity was normalized to protein concentration and expressed in AU. I/R, ischaemia/reperfusion; MMP, matrix metalloproteinase; MLC1, myosin light chain type 1 (ventricular); *P < 0.05 vs aerobic control; ^#^ *P* < 0.05 vs I/R; n = 5‐10

To check if increased expression of mRNA for MMP‐2, MMP‐9 is accompanied by increased protein synthesis, appropriate quantitative ELISA tests were used. Data confirmed an elevated synthesis of MMP‐2 (Figure 5A) in hearts subjected to I/R. Administration of the tested compound into perfused hearts led to reduced tissue expression of MMP‐2 to the level of aerobic control. Tissue content of MMP‐9 was not detectable in any group. We have also observed significantly reduced activity of MMP‐2 according to 10 µmol/L barbiturate in comparison to elevated activity of MMP‐2 in hearts subjected to I/R without potential drug (Figure 5B).

Western blot analysis showed decreased amount of MLC1 in hearts subjected to I/R However, 10 minutes of perfusion of experimental hearts with 10 µmol/L barbiturate before induction of ischaemia and during the first 10 minutes of reperfusion led to decreased tissue injury and MLC1 degradation (Figure 5C). Degradation of MLC1 in the heart tissue negatively correlated with increased activity of MMP‐2 (Figure 5D).

### 3.4 5‐phenyloxyphenyl‐5‐aminoalkyl nitrate barbiturate as a donor of NO

**Figure 6:**
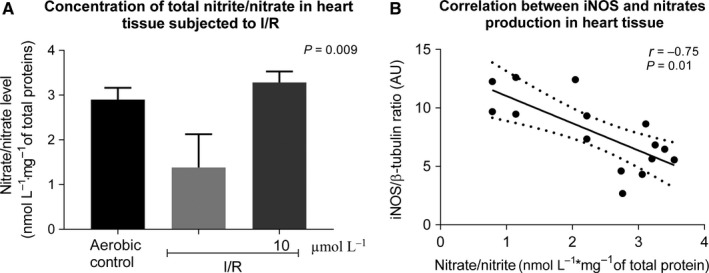
An influence of 5‐phenyloxyphenyl‐5‐aminoalkyl nitrate barbiturate on NO production (A). Correlation between iNOS and NO (B). iNOS, inducible nitric oxide synthase; I/R, ischaemia/reperfusion; *P < 0.05 vs aerobic control; ^#^ *P* < 0.05 vs I/R; n = 5‐10

**Figure 7:**
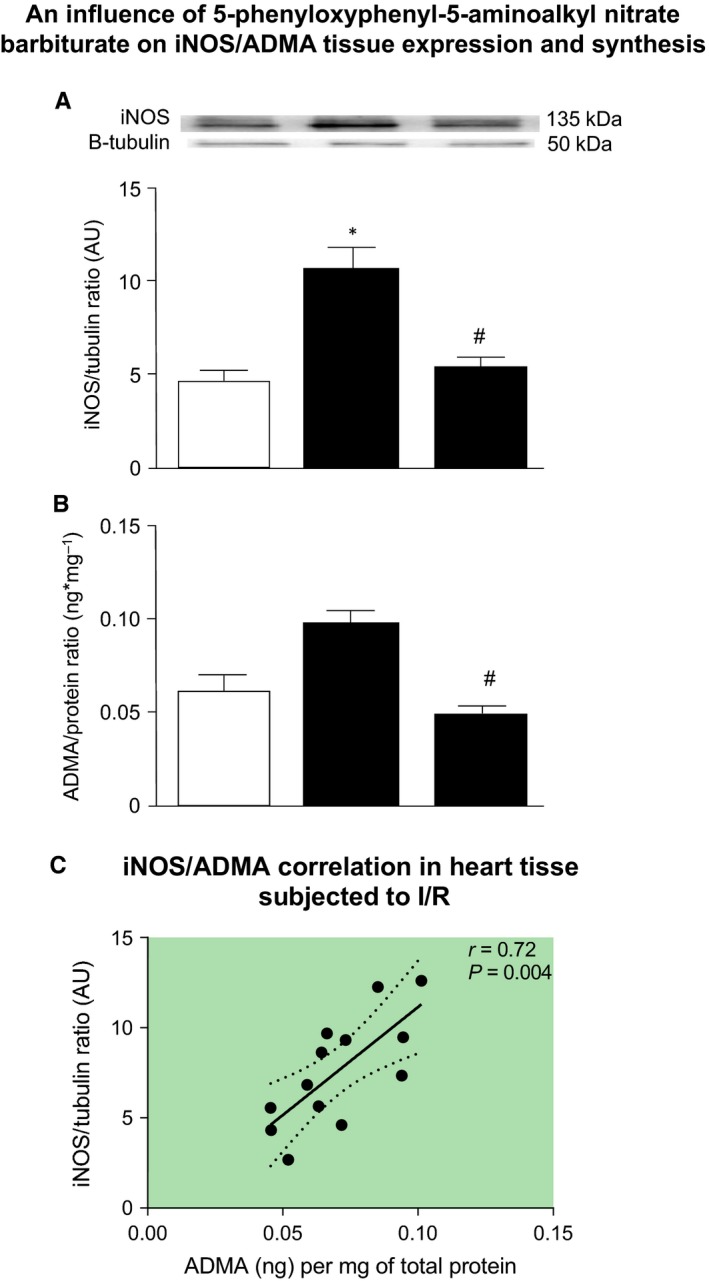
An influence of 5‐phenyloxyphenyl‐5‐aminoalkyl nitrate barbiturate on iNOS (A), ADMA (B) tissue expression and synthesis as well as their correlation (C). ADMA‐asymmetric dimethylarginine; iNOS‐inducible nitric oxide synthase; I/R‐ischaemia/reperfusion; *P < 0.05 vs aerobic control; ^#^ *P* < 0.05 vs I/R; n = 5‐10

The next aim of this study was to evaluate whether an infusion of the hybrid as a donor of NO, into hearts subjected to I/R, may affect the synthesis of inducible form of NOS (iNOS), ADMA. We have observed decreased tissue content of NO (showed as total nitrite/nitrate content) during I/R and an increased tissue NO during perfusion with barbiturates (Figure 6A). An infusion of NO donor also led to significant reduction of iNOS and ADMA, more than 48% and 46% respectively (Figure 7A‐B). The analysis of correlations showed that iNOS positively correlated with production of ADMA (Figure 7C) and iNOS negatively correlated with NO content (Figure 6B).

## 4 DISCUSSION

It is well established that cardiac I/R injury results in microvascular damage or myocardial stunning.^3^, ^27^, ^28^ Although the complete molecular basis for myocardial injury following I/R is not fully understood, it is known that degradation of contractile proteins and inadequate blood supply contribute to the pathogenesis of this injury.^27^, ^28^, ^29^ BG Hughes and R Schulz also proposed that oxidative stress, mediated by a burst of RONS, increased activation of MMP‐2, intracellular and mitochondrial Ca^2+^ overload and opening of the mitochondrial permeability transition pore may play a role in this process.^30^ Therefore, the main aim of the current study was to study if simultaneous inhibition of MMP‐2 activity and supplementation with exogenous NO protects the heart from I/R injury.

Wang et al showed that certain N‐substituted homopiperazine barbiturates are selective and highly potent inhibitors of gelatinases (MMP‐2 and MMP‐9), enzymes critically involved in extracellular matrix remodeling.^31^ The mechanism of inhibition is based on chelation of the catalytic zinc in a tridentate mode directing the 5‐substituents into the S2′ substrate binding pocket of Matrix metalloproteinases (MMPs). The barbiturate class of MMP inhibitors are substituted in such a way that it completely removes their classical sedative effects. Due to their in vivo stability, they are more attractive in comparison to other chemical classes of MMP inhibitors such as hydroxamates. The same group later showed that barbiturate‐based matrix metalloproteinase inhibitors containing NO donor‐mimetic group can attenuate gelatinase secretion and its activity thereby regulating enzyme activity at multiple levels.^23^ These authors also demonstrated that NO release leads to inhibition of MMP‐9 transcription and secretion.^23^ Interestingly, MMP gene expression is dynamically up‐regulated by endothelin‐1, angiotensin II and interleukin‐1 beta and this gene has a functional AP‐1 element required for hypoxia‐induced expression.^30^ The dual function of the barbiturate‐nitrate hybrid prompted us to explore a potential cardioprotective role of this substance during I/R induced injury. Our study provides support for a potential protective effect of 5‐phenyloxyphenyl‐5‐aminoalkyl nitrate barbiturate on myocardium subjected to ischemic insult.

Hypoxic injury of the cardiovascular system is one of the most frequent complications following ischemic state.^32^ The deprivation of oxygen and nutrient supply results in a series of sudden biochemical and metabolic changes within the myocardium leading to cell death.^2^, ^33^ The goal of reoxygenation/reperfusion is to restore the proper oxygen supply in hypoxic tissues to salvage viable myocardium. However, reperfusion leads to an acute increase in oxidative stress, which triggers a cascade of pathophysiological events that can paradoxically induce myocardial injury, thereby mitigating the full benefits of reperfusion in terms of myocardial infarction size reduction.^33^, ^34^ There are several hypotheses regarding the mechanism of I/R injury. Some assume a pivotal role for increased production of ROS^13^ and enhanced post‐translational modifications of proteins which are then more susceptible to proteolytic degradation^29^ and uncontrolled activation of MMPs.^15^

MMPs are a family of more than 25 proteolytic enzymes with similar substrate specificity that play many important roles in a variety of physiological and pathological processes.^35^ MMP‐2 is found in almost all tissue types and degrades collagens as well as other extracellular matrix proteins. Enhanced MMP activity is implicated in pathological states in the cardiovascular system including atherosclerosis, restenosis, ischemic heart disease and heart failure.^35^, ^36^, ^37^, ^38^, ^39^

There are also studies showing that increased activation of MMPs in hearts subjected to I/R is a key factor of impaired mechanical function of the heart^15^ and I/R injury.^8^, ^11^, ^15^, ^29^, ^32^, ^40^, ^41^ As increased degradation of intracellular proteins due to increased expression and/or activation of MMPs may contribute to I/R injury, we evaluated the impact of 5‐phenyloxyphenyl‐5‐aminoalkyl nitrate barbiturate on this injury. We showed increased expression of MMP‐2 gene in human cardiomyocytes as well as an increased cardiac synthesis and activity of MMP‐2 protein during I/R conditions. Moreover, infusion of barbiturate hybrid into rat hearts suppressed MMP‐2 expression and activity. Furthermore, increased tissue expression and synthesis of MMP‐2 was associated with increased tissue damage during I/R This effect of MMP‐2 was prevented by administration of NO donor and MMP inhibitor hybrid that improved heart mechanical function and cardiomyocyte contractility in a concentration‐dependent manner. Improved mechanical function of the heart was the consequence of decreased heart tissue damage. Indeed, we showed that intracellular activity of LDH (the marker of cell injury Ref. 42) in hearts subjected to I/R in the presence of tested barbiturate was greater than in I/R control, an effect attenuated by the tested compound.

In 2005, Sawicki et al reported that MLC1 is yet another intracellular target for MMP‐2 proteolytic activity in I/R hearts.^12^ Our study also confirmed that MLC1 (ventricular isoform of myosin light chain) was highly degraded during I/R and this degradation correlated with increased activity of MMP‐2, confirming the mechanism of hearts injury. A direct inhibitory effect of the hybrid on MMP‐2 in combination with inhibition of signalling pathway for MMP‐2 expression caused decreased proteolysis of MLC. MLC1 is a structural component of the thick filament of the sarcomere. As an element of sarcomere it is responsible for proper heart contractile function.^43^ Therefore, inhibition of MMP‐2 activity and MLC proteolysis could be the main causation of improved heart function in our model.

**Figure 8:**
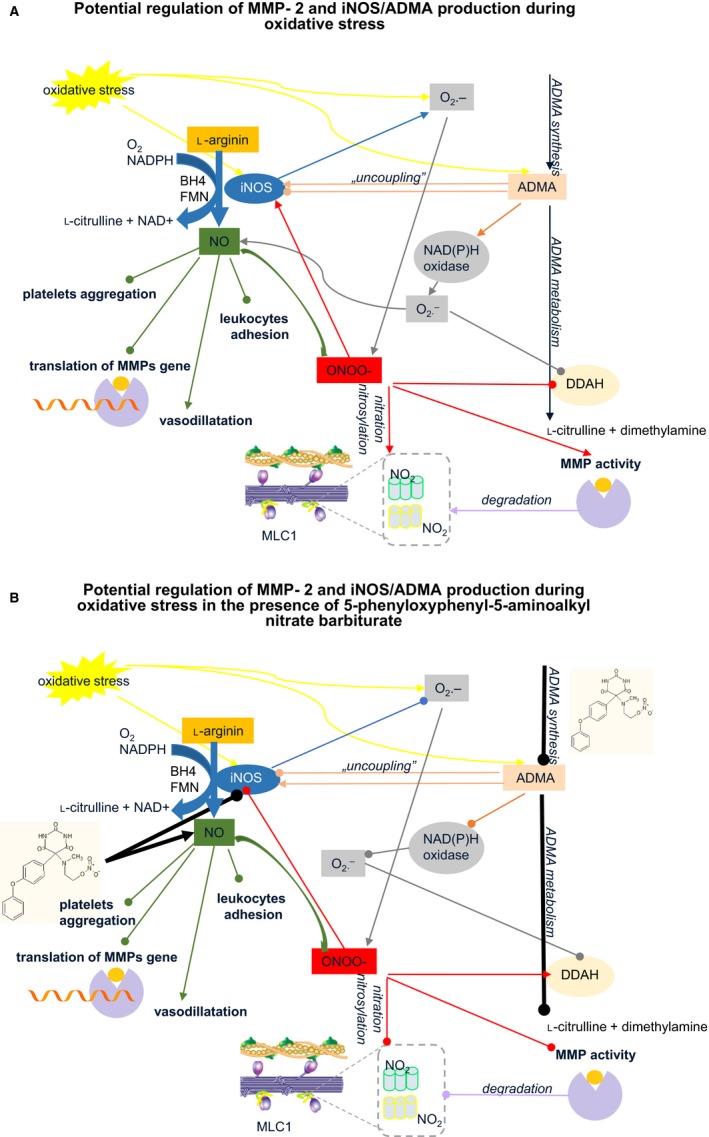
Potential regulation of MMP‐2 and iNOS‐ADMA pathway during oxidative stress in the absence (A) and the presence (B) of 5‐phenyloxyphenyl‐5‐aminoalkyl nitrate barbiturate‐ the scheme. NO is produced by inducible form of nitric oxide synthase (iNOS) from l‐arginine in the presence of oxygen, NADPH (nicotinamide adenine dinucleotide phosphate), BH4 (tetrahydrobiopterin) and FMN (flavin mononucleotide) and plays a crucial role in vasodilatation (increasing coronary flow‐CF), leukocytes adhesion, platelets aggregation and MMP‐2 expression. Oxidative stress leads to generation of ROS induced iNOS expression and production of endogenous asymmetric dimethylarginine (ADMA). ADMA in turn enhanced ‘iNOS uncoupling’ generating large amounts of $O_{2}^{-}$·, which in turn rapidly reacts with NO leading to peroxynitrite production (ONOO^−^). ONOO^−^ plays multiple roles such as: inhibition of ADMA metabolism by DDAH, activates MMP‐2 and leads to nitration/nitrosylation of myosin light chains (MLC1s). Arrows mark the stimulating effect (→), lines terminated with a circle means an inhibition (‐o)

It has been well established that the restoration of blood flow to the myocardium subjected to I/R generates oxidative stress due to an increased formation of reactive oxygen species (ROS) in the ischemic area.^1^, ^44^, ^45^ Oxidative stress also triggers the increased expression of inducible NO synthases (iNOS) and subsequent production of nitric oxide (NO) and peroxynitrite (ONOO^−^),^13^, ^16^ which in turn leads to the activation of MMP‐2 and heart damage.^6^ Inducible NOS is not usually expressed in cells, but its expression can be induced by bacterial lipopolysaccharide, cytokines and other agents including hypoxia.^46^ Once expressed, iNOS is constantly active producing NO until the enzyme is degraded as iNOS; production of NO is not regulated by intracellular Ca^2+^ concentration.^47^ Increased amounts of NO thus generated react with superoxide (O_2_) and form ONOO^−^, thereby limiting NO production^48^ and increasing MMP‐2 activity and decreasing DDAH activity^13^ (Figure 8A). Increased elimination of NO through reaction with O_2_·makes NO unavailable to regulate smooth muscle. In addition, iNOS may become uncoupled and produce ROS leading to conversion of iNOS from an NO producing enzyme to an enzyme that generates $O_{2}^{-}$.^21^ Our study confirmed significantly increased tissue expression of iNOS in hearts subjected to I/R, however we have observed decreased production of NO (as total nitite/nitrate) in those hearts. It is also widely documented that reduced NO availability is considered an important risk factor for acute cardiovascular events.^27^, ^49^ Interestingly, infusion of MMP‐2‐inhibitor‐NO‐donor hybrid led to reduced iNOS synthesis and increased tissue content of NO. This supports the hypothesis that NO is effectively released by the tested compound.

Asymmetric dimethylarginine (ADMA) is an l‐arginine analog that serves as an endogenous inhibitor of isoforms of NOS.^18^, ^50^, ^51^, ^52^ ADMA has been reported to competitively inhibit NO synthesis by displacing l‐arginine from NOS.^53^ This study confirmed an increased ADMA expression and production in hearts subjected to I/R (Figure 6). As the l‐arginine/NO pathway actively participates in the regulation of numerous physiological processes including vasodilation,^54^ inhibition of platelet aggregation^55^ and leukocyte adhesion,^56^ an enhanced ADMA synthesis and decreased NO production could contribute to enhanced tissue damage. Our observations are consistent with other studies, including both animal models and patients, in which enhanced ADMA levels correlated with pulmonary hypertension, hypercholesterolemia and endothelial dysfunction and atherosclerosis.^57^, ^58^, ^59^ Accumulation of ADMA would be expected to enhance cardiovascular disorders through loss of NO.^17^ Moreover, studies in cultured human endothelial cells have shown that elevated ADMA results in the production of $O_{2}^{-}$. As described above, ADMA may uncouple NOS resulting in a switch of the enzymatic activity from NO to ROS production. In turn, an increased production of $O_{2}^{-}$·leads to an inhibition of *N* ^G^,*N* ^G^‐dimethylarginine dimethylaminohydrolase (DDAH), a key enzyme that metabolizes ADMA and enhanced production of toxic peroxynitrite, which activates MMPs^13^ and modifies contractile proteins^32^ (Figure 8A). Interestingly, administration of nitrate hybrid compound into hearts undergoing I/R led to decreased production of ADMA, probably as a consequence of decreased tissue expression of iNOS and supply of exogenous NO (Figure 8B).

In conclusion, this study confirms the potential, dual cardioprotective role of an MMP‐inhibitory nitrate hybrid compound in the development of I/R injury. We showed that the hybrid compound can suppress MMP‐2 mRNA expression and activity, hence limiting injury. Moreover, this compound decreases iNOS and ADMA production thus normalizing the NO bioactivity. Therefore, 5‐phenyloxyphenyl‐5‐aminoalkyl nitrate barbiturate shows cardioprotective activity and this pharmacological profile could be further explored in pre‐clinical and early‐clinical investigations.^60^

## CONFLICT OF INTEREST

The authors have no conflicts of interest to disclose.
